# Fermented rice germ extract with high concentration of gamma-aminobutyric acid enhances pentobarbital-induced sleep via GABAergic system in rats

**DOI:** 10.1371/journal.pone.0326859

**Published:** 2025-08-12

**Authors:** Minsook Ye, Woojin Jeong, Hyo Jeong Yu, Kyoung-min Rheu, Bae-jin Lee, Insop Shim

**Affiliations:** 1 Department of Physiology, College of Medicine, Kyung Hee University, Seoul, Republic of Korea; 2 Marine Bioprocess Co., Ltd., Busan, Republic of Korea; Zhengzhou University, CHINA

## Abstract

In modern societies, insomnia stands out as a widespread health concern. Gamma-aminobutyric acid (GABA), an inhibitory neurotransmitter, can exert hypnotic effects. However, despite various clinical trials conducted, scientific evidence regarding the impact of ingested GABA on sleep remains unclear. Therefore, in this study, we investigated the sedative effects of fermented rice germ (RG30) extract with a high GABA concentration through electroencephalography (EEG) analysis in a pentobarbital-induced sleep animal model. The exploration into the neural basis of these positive effects involved evaluating orexin, GABA_A_ receptor, and serotonin (5-HT) immunoreactivity in the brain using immunohistochemical methods. Furthermore, we conducted a binding assay for GABA_A_ receptors and 5-HT_2C_ receptors, as these are considered pivotal targets in the mechanism of action for sleep aids. In the binding assay, RG30 displayed binding affinity to the GABA_A_ receptor (IC50 value of 5.911 µg/mL) and 5-HT_2C_ receptor (IC50 value of 56.89 µg/mL). Administration of RG30 increased NREM sleep time and reduced wake time in a pentobarbital-induced sleep model. Low-dose RG30 (RG30_L; 40 mg/kg) induced significant alterations in EEG-defined sleep architecture, whereas high-dose RG30 (RG30_H; 90 mg/kg) predominantly modulated orexin expression in the lateral hypothalamus and GABA_A_ receptor levels in the ventrolateral preoptic nucleus. No significant changes were observed in 5-HT expression within the dorsal raphe nucleus. In conclusion, RG30 increased NREM sleep and significantly improved sleep behavior due to its action on the GABA_A_ receptors. The collective findings suggest that RG30 could be considered as a novel sleep-enhancing agent in pharmaceutical and food industries.

## Introduction

Insomnia is a common sleep-related complaint, affecting approximately 10–40% of adults. Moreover, 5–10% of the adult population experiences chronic insomnia, with a higher prevalence among those with concurrent medical or psychiatric illnesses [1]. Drug classes commonly used for the treatment of insomnia include gamma-aminobutyric acid type A (GABA_A_)-benzodiazepine (BZD) receptor agonists, melatonin receptor agonists, antidepressants, and antihistamines. However, these medications have associated side effects, and efforts to identify an ideal sedative-hypnotic agent that provides better results without side effects are ongoing. In this context, natural sleep aids, which can improve sleep quality without side effects, are increasingly used as alternatives [2].

GABA is an inhibitory neurotransmitter that plays a crucial physiological role in humans by reducing neuronal activity, regulating heart rate, enhancing memory, and alleviating sleep disorders [3]. Three pharmacologically distinct classes of GABA receptors—GABA_A_, GABA_B_, and GABA_C_—have been delineated. GABA_A_ receptors function as ligand-gated chloride ion (Cl^−^) channels, responding to GABA and its agonist, muscimol, while being antagonized by bicuculline. Moreover, they are intricately associated with various binding sites, including GABA, barbiturates, BZDs, and picrotoxins. GABA_A_ receptors can swiftly impede neurotransmission and contribute to tonic inhibition; by this means, they are crucial therapeutic targets for insomnia [4]. BZDs can stimulate the opening of Cl^−^ influx using GABA, thereby inhibiting neurotransmission. These drugs share common pharmacological properties, such as sedative-hypnotic, anxiolytic, and anticonvulsant effects.

Numerous recent studies in human have suggested that foods enriched in GABA, such as rice and tea, possess stress-reducing properties [5]. Notably, the consumption of GABA-enriched rice has been associated with decreased anxiety scores and blood cortisol levels [6]. Fermentation has been observed to contribute to increased GABA content in diverse natural sources. Previous studies have indicated that using *Lactobacillus brevis* BJ 20 (*L. brevis* BJ20) in fermentation notably increases GABA levels in oyster extracts and seaweed [7]. The fermentation of seaweed and oyster by *L. brevis* BJ20 enhanced both antioxidant and anti-inflammatory activities, promoting stress relief and sleep enhancement [8].

Previous studies have demonstrated the sleep-improving effects of gamma-aminobutyric acid (GABA) derived from fermented rice germ (RG). GABA extracted from fermented RG has been shown to alleviate caffeine-induced sleep disturbances, including delayed sleep onset and reduced sleep duration, in murine models [9]. In addition, a randomized, double-blind clinical trial reported that fermented RG extract containing GABA significantly decreased sleep onset latency and enhanced sleep efficiency in individuals with insomnia [10]. Previous studies have demonstrated the sleep-improving effects of gamma-aminobutyric acid. These findings support the potential of fermented RG as a natural sleep aid through GABAergic modulation. Nevertheless, the detailed mechanisms underlying its sedative effects have not been fully elucidated. Therefore, the present study aimed to evaluate the sleep-promoting effects of fermented RG extract with a high concentration of GABA and to investigate its potential mechanisms using a pentobarbital-induced sleep model and electroencephalographic analysis in rats. Another study demonstrated that orally administered GABA significantly increased the alpha wave activity and decreased the beta wave activity in the participants’ brains for 60 min, indicating the neurotransmitter’s ability to promote relaxation and reduce anxiety. Orally delivered GABA, which peaks in the bloodstream 30 min after ingestion, markedly shortens sleep onset time and increases non-rapid eye movement (NREM) sleep time [11]. In clinical studies and animal experiments, RG has demonstrated efficacy in enhancing sleep quality. However, studies investigating the impact of RG30 on sleep are lacking, and there is currently no conclusive understanding of the mechanisms for treating or improving sleep and associated disorders. For this reason, RG30 has been chosen as an intervention to explore its effects on sleep and its association with the GABAergic mechanism.

In this study, we assessed the potential of RG30 as a new therapy for alleviating sleep disturbances using a pentobarbital-induced sleep-related rat model. We aimed to demonstrate the effects of RG30 using a GABA_A_ receptor-binding assay and confirm its sleep-improving effects using animal sleep model experiments.

## Materials and methods

### Preparation of RG30

Raw rice germ, containing glutamic acid as a precursor of GABA, was purchased from Hwasung Energy Co., Ltd. (Gyeongsan, Republic of Korea), and manually washed three times with water before draining. The washed RG was then sent to Marine Bioprocess Co., Ltd. (Busan, Republic of Korea), where it was mixed with water in a 1:10 (w/v) ratio and subjected to enzymatic hydrolysis. For enzymatic treatment, the RG mixture was heated to 60 ± 2°C and treated with a commercial amylase (Ban® 480 L, FG, Novozyms Korea Co., Ltd., Seoul, Republic of Korea) at a concentration of 1% (w/w, based on wet RG weight). After hydrolysis, the RG was autoclaved at 90°C for 30 min and then at 121°C for 10 min to ensure the inactivation of amylase. The filtered hydrolysate was combined with a fermentation medium containing yeast extract (1.5%, w/v), glucose (0.5%, w/v), monosodium glutamate (8%, w/v), and L-glutamic acid (24%, w/v) as nutrients, using the RG hydrolysate as 50% of the total medium volume, and adjusted with distilled water to reach 100%. This mixture was sterilized by autoclaving at 121°C for 15 min and then cooled to room temperature. Lactobacillus brevis BJ-20 (Accession No. KCTC11377 BP) was used as the fermenting strain. A seed culture was prepared by inoculating the strain into a sterilized medium containing yeast extract (3%), glucose (1%), monosodium glutamate (1%), and water (95%), and incubating at 37°C for 24 h. The fermentation was initiated by inoculating the main medium with 10% (v/v) of this seed culture. The fermentation was carried out at 37 ± 1°C for 72 h under static conditions. During fermentation, the glutamic acid from both the RG hydrolysate and supplemented medium was converted to GABA. After fermentation, the culture was filtered using a disk separator (CLARA 200; Alfa Laval, Lund, Sweden). Finally, the sterilized liquid was spray-dried to produce the powdered form of RG.

### High-Performance Liquid Chromatography (HPLC) analysis

#### Chemicals and reagents.

GABA and sodium acetate (50 mM, pH 6.5) were obtained from Sigma-Aldrich (St. Louis, MO, USA). HPLC-grade acetonitrile, methanol, and distilled water (DW) were procured from Samchun Pure Chemical Co., Ltd. (Pyeongtaek, Republic of Korea). The hydrochloric acid solution was provided by Biosesang (Seongnam, Republic of Korea). Borate buffer (0.4 N in water, pH 10.2; Agilent P/N 5061−3339) and o-phthaldialdehyde reagent (10 mg/mL, Agilent P/N 5061−3335) were sourced from Agilent Technologies (Palo Alto, CA, USA). Acetic acid was acquired from Junsei Chemical Co. Ltd. (Tokyo, Japan).

#### Standard solution and sample preparation.

A standard stock solution was prepared by dissolving 0.1 g of GABA in 100 mL of distilled water using a volumetric flask. The solution was then filtered through a 0.2 μm polytetrafluoroethylene (PTFE) syringe filter (25 mm, Merck Millipore, MA, USA). The resulting standard solution was aliquoted and stored at –80°C until analysis. A calibration curve was constructed using serial dilutions of the stock solution to cover a concentration range of 62.5–500 μg/mL.

#### HPLC analysis method.

A Dionex U3000 series HPLC system (Thermo Fisher, Waltham, MA, USA) equipped with a UV detector was used, and the flow rate was reduced to 1 mL/min. The samples were analyzed using UV–Vis spectrophotometry at a wavelength of 338 nm. The GABA content was calculated using the peak area corresponding to GABA in the sample chromatogram, referenced against the standard calibration curve. The final concentration (mg/g) was determined using the following equation:


$$\;GABA(mg/g)\;=\;\left(\frac{Concentration\;from\;calibration\;curve\;\left(\frac{mg}{mL}\right)\;\times \;Dilution\;volume\;(mL)}{Sample\;weight\;(g)}\right)\mathrm{\backslash ]}$$

Based on this method, the GABA content of the fermented rice germ extract (RG30) was determined to be 31.04 mg per 100 mg, equivalent to 31.04% (w/w) of the dried powder.

### 5-HT2c receptor binding assay

The 5-HT2c receptor (serotonin 5HT2c membrane preparation in HEK293 cells) was procured from PerkinElmer (Waltham, MA, USA), and the GABA receptor was sourced from rat brain tissue. The Protein Chip was obtained from Proteogen, and Cy5-labeled tryptamine and Cy5-labeled muscimol were acquired from Peptron. The GABA and 5-HT2c receptors stock buffer comprised 50 mM Tris-HCl (pH 7.4), 0.5 mM EDTA, 10 mM MgCl_2_, and 10% sucrose. The GABA and 5-HT2c receptor-binding assay buffer comprised 50 mM Tris-HCl, 10 mM MgCl_2_, 1 mM EDTA, and 0.1% BSA at pH 7.4. The 5-HT2c receptor (50 µg/mL) was immobilized on the Protein Chip, serving as a substrate to capture protein, for 16 h at 4°C. After double washing in 0.05% phosphate-buffered saline containing 0.2% Triton X-100 (PBST) for 10 min and drying with nitrogen gas (N_2_), the Protein Chip underwent a 1-h blocking step at room temperature using 3% BSA. Following three washes with PBST and drying, Cy5-labeled tryptamine (500 µM, with 30% glycerol in PBS as a buffer) and RG (using 30% glycerol in PBS as a buffer) were applied to the Protein Chip and incubated for 1 h at 37°C. Subsequently, the Protein Chip was rinsed with PBST and DW and dried under a stream of N_2_ gas. RG, dissolved in ethanol and diluted to the desired concentration using PBS, covered a concentration range from 1000 µM to 15.625 µg/mL. Tryptamine and muscinol were used as negative controls. RG was used to evaluate the sedative effect of the GABA receptor and 5-HT_2c_ receptor binding assays.

### Animals

Adult male Sprague–Dawley rats were obtained from Samtako (Osansi, Gyeonggi-do, Republic of Korea). These animals were accommodated in a climate-controlled environment, with temperature maintained at 20–25°C and humidity level at 45–65%, following a 12-h light and 12-h dark cycle (lights on at 8 a.m.). Food and water were provided ad libitum throughout the study. The laboratory animals were handled and treated in compliance with the guidelines outlined by the Ministry of Food and Drug Safety National Institute of Toxicological Research, per the standards for laboratory animal care and usage. This experiment was approved by the Institutional Animal Care and Use Committee of Kyung Hee University (KHUAP(SE)-14–051). Rats were randomly assigned to five groups: untreated, naïve (Nor, n = 8), pentobarbital injected with vehicle (CON, n = 9), pentobarbital injected along with 40 mg/kg RG (RG_L, n = 5), pentobarbital injected along with 90 mg/kg RG (RG_H, n = 7), and pentobarbital injected along with 10 mg/kg DZP (DZP, n = 7). Diazepam is widely recognized as a sedative drug and was used as a positive control in this experiment. The number of animals assigned to each group differed according to the specific experimental procedures performed. In particular, electroencephalographic (EEG) recording involved surgical implantation of electrodes and required a post-operative recovery period, thereby limiting the number of animals applicable for this procedure. Accordingly, the number of animals per group was determined based on methodological requirements and ethical considerations.

### EEG surgery

The subjects were divided into five groups: normal (Nor; n = 8), control (Con; n = 9), low-dose RG-treated (RG_L; n = 5), high-dose RG-treated (RG_H; n = 7), and diazepam-treated (DZP; n = 7). Electroencephalogram (EEG) electrodes were surgically implanted to facilitate polygraphic recordings, following the guidelines delineated in the Paxinos and Watson stereotaxic atlas [12]. Surgical anesthesia was induced with intraperitoneal pentobarbital (40 mg/kg). Subsequently, the rats were subjected to chronic implantation of a head mount. The transmitter body was subcutaneously placed off the midline, posterior to the scapula, secured to the skin, and stabilized using three sutures. Skull-mounted electrodes were secured with screws and dental cement. All surgical interventions were performed using a stereotaxic methodology in an aseptic environment. Postoperatively, each rat was allowed a 7-day recovery period in separate transparent enclosures.

### Methodology of EEG recording

Following the recovery phase, the rats were acclimated to the recording settings before testing. RG_L, RG_H, and DZP were prepared in 0.9% saline (RG_L concentration = 40 mg/kg, RG_H concentration = 90 mg/kg, DZP concentration = 10 mg/kg) and orally administered for five consecutive days before the EEG recording commenced. The oral administration of saline, RG_L, RG_H, and DZP was performed 10 min before the EEG recording sessions. After treatment, animals were immediately connected to EEG recording cable (two EEG channels). The software indicates wakefulness as high-frequency low-amplitude EEG, and NREM was scored on the presence of spindles scattered with slow waves in the EEG. EEG power during REM was significantly decreased in lower-frequency δ-wave (0.75–4 Hz) and increased in the range of θ-wave activity (5.0–9.0 Hz, peak at 7.5 Hz).Rats were habituated to the recording conditions prior to the test after operation. The recordings were initiated at 8:00 p.m., capturing 12 h of EEG recordings and activity in all subjects. The cortical EEG signals were amplified (x100), filtered through a low-pass filter at 100 Hz, digitized at a sampling rate of 200 Hz, and recorded using a PAL-8200 data acquisition system from Pinnacle Technology Inc. The recordings were performed at a chart speed of 25 mm/s.

### EEG data analysis

The SleepSign Ver. 3 software (Kissei Comtec, Nagano, Japan) automatically classified sleep–wake states into three categories: wakefulness (Wake), rapid eye movement (REM) sleep, and NREM sleep. Sleep latency was defined as the duration from the administration of the sample to the onset of the initial uninterrupted NREM sleep episode, lasting a minimum of 2 min and not interrupted by more than six 4-s epochs that were not scored as NREM sleep.

### Measurement of corticosterone concentration in the serum

After the behavioral tests, rats were euthanized after being anesthetized with pentobarbital. Cardiac blood was collected and centrifuged to separate the serum. The obtained samples were stored at −80°C until the enzyme-linked immunosorbent assay (ELISA kit; Enzo Life Sciences, New York, USA) was performed to measure serum corticosterone.

### Immunohistochemistry

After transcranial perfusion, the rat brains were removed with 4% formaldehyde solution (Sigma-Aldrich Co.), post-fixed in the same fixative for 24 h, and placed in phosphate-buffered saline containing 20% sucrose for 72 h. Serial 30 µm-thick coronal sections were cut using a cryostat microtome (CM1850UV; Leica Microsystems Inc., Wetzlar, Germany) and stored at −20°C; they were histochemically processed as free-floating sections. Sections were washed three times with PBST.

Primary rabbit polyclonal antibodies against orexin (Abcam, Cambridge, MA, USA) were diluted to 1:800. Antibodies targeting the GABA_A_ receptor (Abcam, Cambridge, MA, USA) and 5-HT (Millipore, Cambridge, MA, USA) were diluted to 1:800. The sections underwent a 12-h incubation at 4°C with constant agitation. After rinsing with PBST, a 2-h incubation at room temperature was performed using a biotinylated goat anti-rabbit antibody (Vector Laboratories, Inc., Burlingame, CA, USA) diluted to 1:200 in PBST with 2% v/v normal goat serum. Subsequently, the sections were exposed to an avidin-biotin-peroxidase complex reagent (Vector Laboratories) for 2 h at room temperature. After further rinsing with PBST, the tissues were developed using a DAB substrate kit (Vector Laboratories). The final steps included mounting the sections on slides, air-drying, and covering them for microscopic observation.

### Statistical Analysis

All statistical analyses were conducted using SPSS (IBM^Ⓡ^ SPSS^Ⓡ^ Statistics Ver. 23 Chicago, IL, USA). For multiple comparisons, behavioral data were analyzed using one-way analysis of variance (ANOVA) and t-tests. Tukey’s post-hoc test was used to identify significant differences among groups. The level of significance was set at **p* *< 0.05*.*

## Results

### GABA content in RG

**Fig 1:**
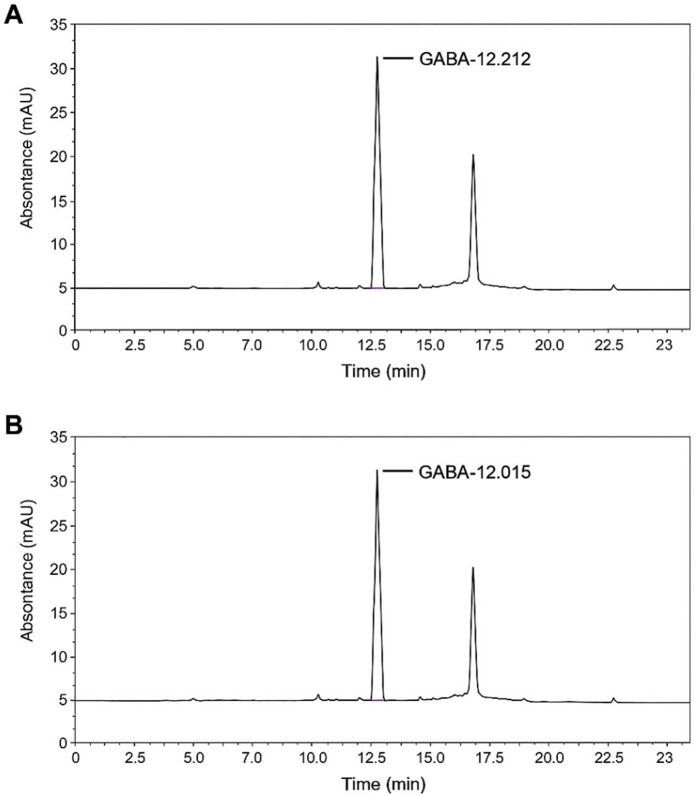
RG chemical analysis. HPLC chromatograph of GABA standard (A) and GABA in RG **(B)**.

We performed HPLC analysis to determine the GABA content in RG. The presence of GABA after fermentation was confirmed by comparing the retention times of the standard and RG (Fig 1). Furthermore, HPLC chromatography showed that the average percent content of GABA in RG was 31.04 ± 0.94%.

### Binding affinity of RG to GABA_A_ and 5-HT_2C_ receptors

**Fig 2:**
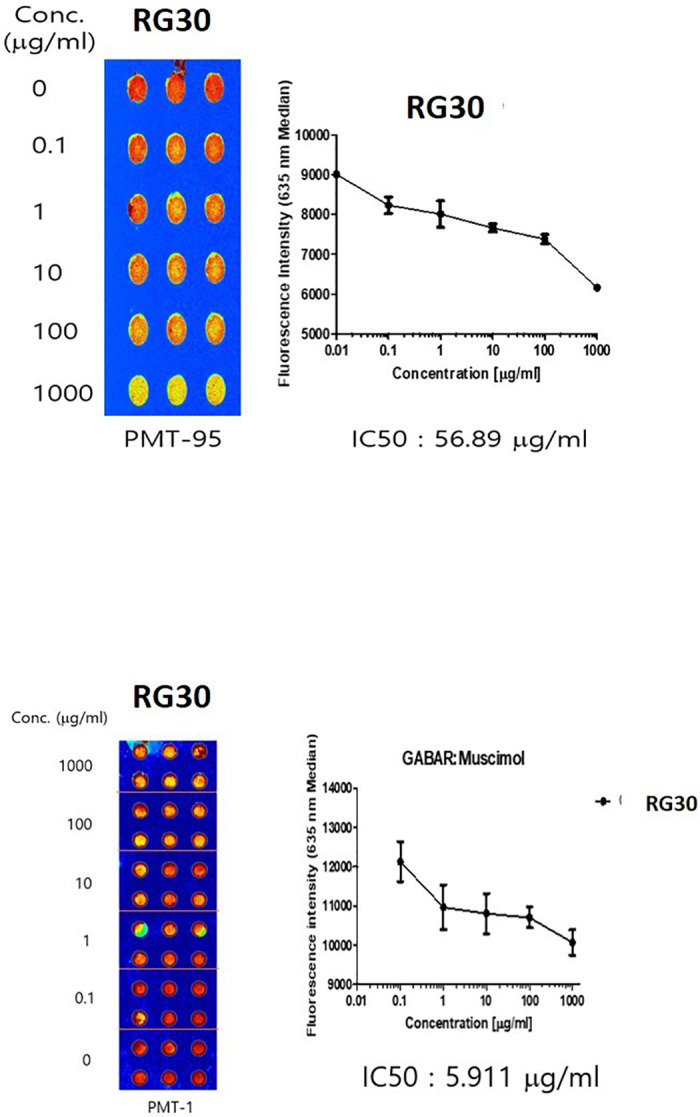
Screening of GABA_A_ and 5-HT_2C_ receptor antagonists from RG using a protein chip-based GABA_A_ and 5-HT_2C_ receptor assay. **(A)** Dose-response curve and IC50 of RG in the GABA receptor binding assay. RG was added to GABA receptors in a dose-dependent manner, revealing an IC50 value of 5.911 µg/mL. The concentrations of GABA_A_ receptors and Cy5-labeled muscinol were 50 μg/mL and 500 μM, respectively. **(B)** Dose-response curve and IC50 of RG in the 5-HT_2C_ receptor binding assay. RG was added to 5-HT_2C_ receptors in a dose-dependent manner, revealing an IC50 value of 56.89 µg/mL. The concentration of 5-HT_2C_ receptors was 50 μg/mL, and the concentration of Cy5-labeled Tryptamine was 500 μM.

RG30 exhibited robust binding activity to the GABA_A_ receptor, characterized by its efficacy as a potential GABA_A_ receptor agonist or allosteric modulator. Notably, the inhibitory concentration (IC₅₀) of RG30 was determined to be 5.911 µg/mL (Fig 2A), indicating a high affinity for the GABA_A_ receptor, which may underlie its anxiolytic-like effects observed in behavioral studies. In addition, RG30 showed binding activity to the 5-HT_2C_ receptor, with an IC₅₀ value of 56.89 µg/mL (Fig 2B). This suggests that RG30 may also modulate serotonergic signaling, further contributing to its potential neuroactive properties.

### Effect of RG on the EEG sleep architecture and profile

**Fig 3:**
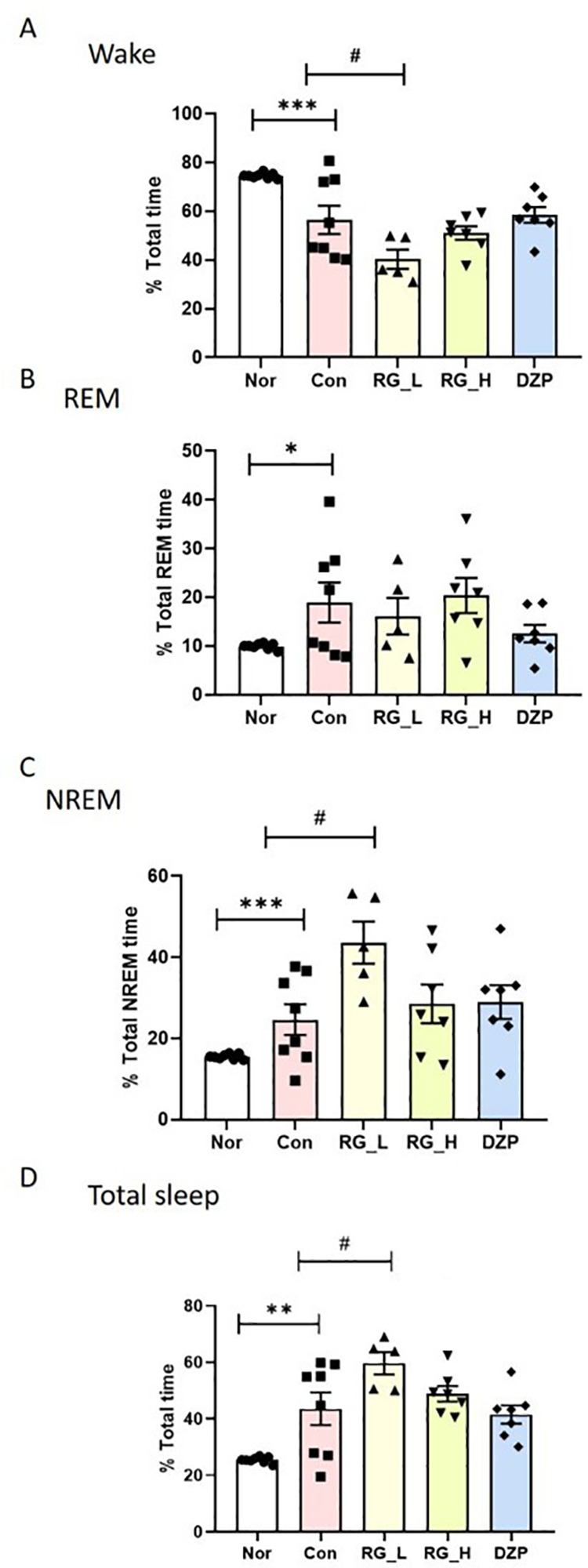
Effects of RG30 on sleep architecture in rats during the dark phase. Changes in the percentage of wake **(A)**, REM sleep **(B)**, NREM sleep **(C)**, and total sleep (D) are shown. Data are expressed as mean ± SEM. Statistical comparisons were performed using one-way ANOVA followed by Tukey’s post hoc test. ***p < 0.001, **p < 0.01, *p < 0.05 vs. Nor group;. #p < 0.05 vs. Con group. Group sizes: Nor (n = 8), Con (n = 8), RG30_L (n = 5), RG30_H (n = 7), DZP (n = 7).

The effects of RG on the EEG sleep architecture and profiles were investigated. In the Con group, there was a significant reduction in wake time (*p* < 0.001; Fig 3A) and a marked increase in REM (*p* < 0.05; Fig 3B), NREM (*p* < 0.001; Fig 3C), and total sleep (*p* < 0.01; Fig 3D) compared with the Nor group. Both RG30_L and RG30_H groups exhibited significant reductions in wake time and increases in NREM and total sleep compared to the control group (p < 0.05). No significant differences were observed between the two RG30 doses. Notably, the DZP-treated group showed a higher wake time and lower total sleep time than both RG30-treated groups.

### Effect of RG30 on the number of orexin-positive cells in the Lateral Hypothalamus (LH)

**Fig 4:**
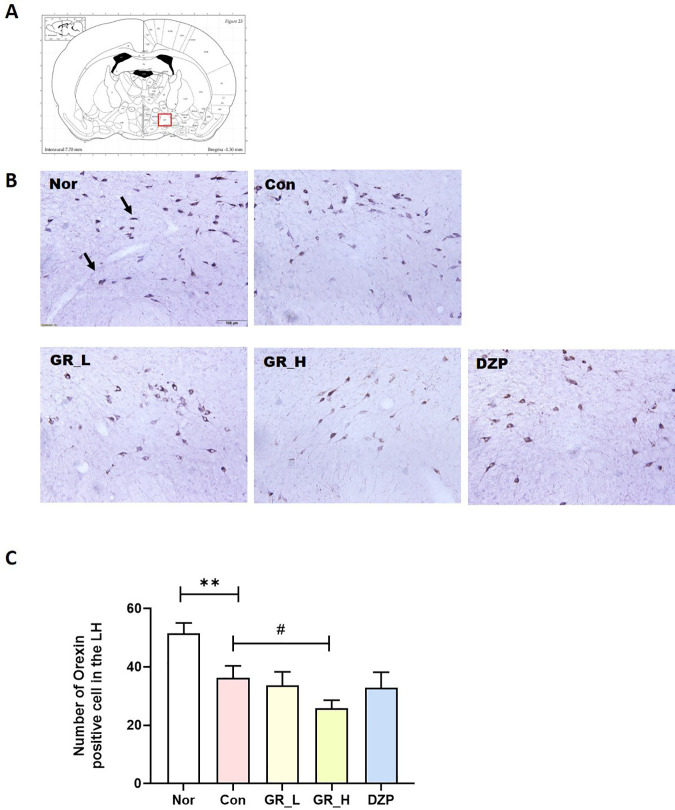
Impact of RG30 on orexin-positive cells in the LH. **(A)** Location of the LH and red box indicate the LH **(B)** Photomicrographs illustrating orexin-positive cells in the LH. **(C)** Quantification of orexin-positive cells in the LH. **p < 0.01 vs. Nor; #*p* < 0.05 vs. Con; one-way ANOVA followed by Tukey’s test. Nor (n = 25), Con (n = 25), RG30_L (n = 19), RG30_H (n = 28), DZP (n = 30). The scale bar represents 100 µm.

Orexin neurons within the LH are pivotal in governing the modulation of sleep–wake patterns. Pentobarbital administration induces sleep by exerting inhibitory effects on the central nervous system, resulting in diminished orexin activity in the LH. We examined orexin expression in the LH (Fig 4) and found that the number of orexin-positive cells was lower in the Con group than in the Nor group (*p* < 0.001; Fig 4). The RG30_H group showed a dramatic reduction in the number of orexin-positive cells compared with the Con group.

### Effect of RG30 on the number of GABA_A_ receptor-positive cells in the Ventrolateral Preoptic Nucleus (VLPO)

The VLPO plays a pivotal role in regulating sleep. GABAergic neurons predominantly act via GABA_A_ receptors and are concentrated in the VLPO. Pentobarbital administration augments GABA_A_ receptor activity in the VLPO.

**Fig 5:**
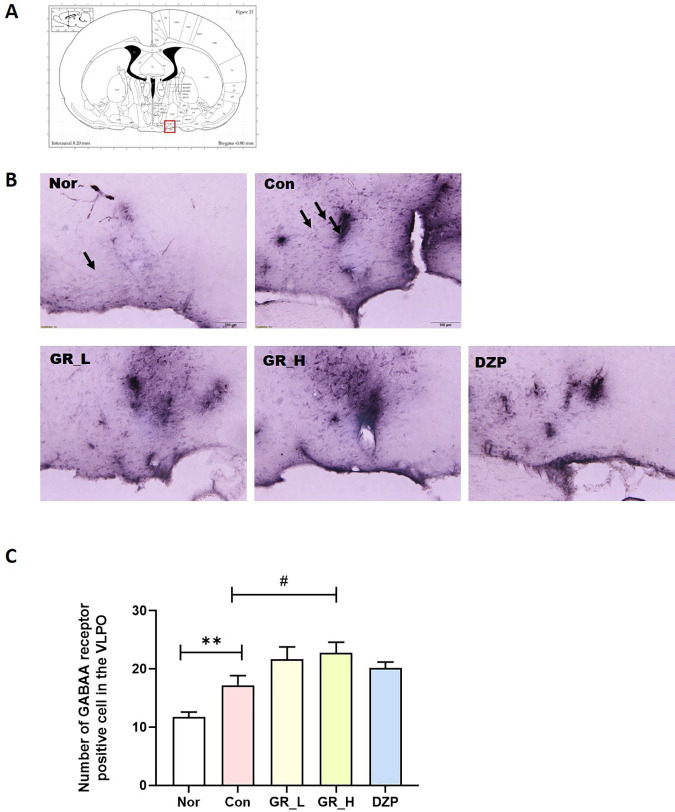
Effects of RG30 on GABA_A_ receptor-positive cells in the VLPO. **(A)** Location of the VLPO and red box indicate the VLPO **(B)** Photomicrographs illustrating GABA_A_ receptor-positive cells in the VLPO. **(B)** Quantification of GABA_A_ receptor-positive cells in the VLPO. **p < 0.01 vs. Nor; #*p* < 0.05 vs. Con; one-way ANOVA followed by Tukey’s test. Nor (n = 30), Con (n = 36), RG30_L (n = 27), RG30_H (n = 29), DZP (n = 46). The scale bar represents 100 µm.

The expression of GABA_A_ receptors was measured in the RG30-treated groups. We measured GABA_A_ receptor expression in the VLPO (Fig 5) and found that the number of GABA_A_ receptors-positive cells was higher in the Con group than in the Nor group (*p* < 0.01; Fig 5). The RG30_H group showed a dramatic reduction in the number of GABA_A_ receptor-positive cells compared with the Con group (*p* < 0.05; Fig 5).

### Effect of RG30 on the 5-HT level in the Dorsal Raphe Nucleus (DRN)

**Fig 6:**
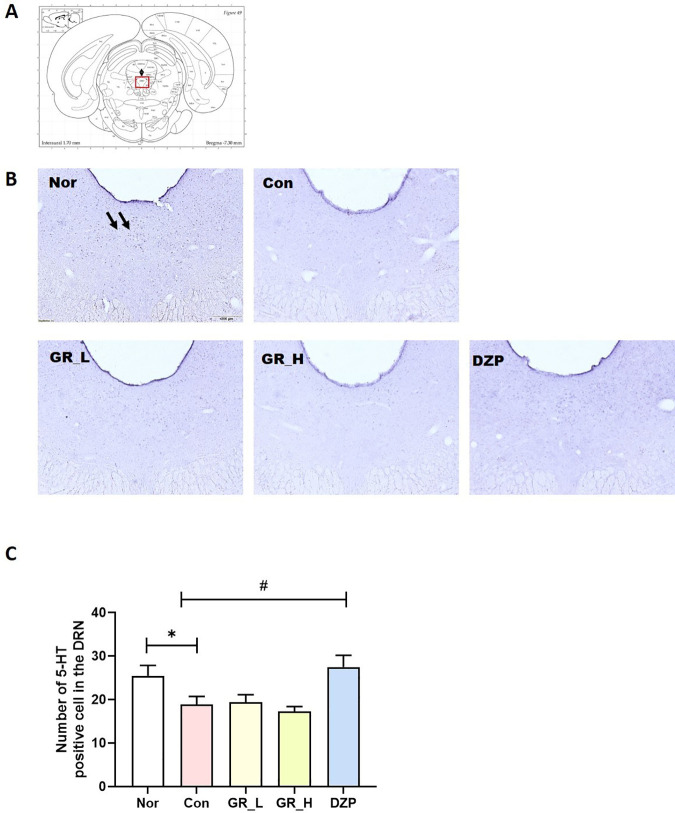
Effects of RG30 on 5-HT-positive cells in the DRN. **(A)** Location of the DRN and red box indicate the DRN **(B)** Photomicrographs illustrating 5-HT-positive cells in the DRN. **(B)** Quantification of 5-HT-positive cells in the DRN. *p < 0.05 vs. Nor; #*p* < 0.05 vs. Con; one-way ANOVA followed by Tukey’s test. Nor (n = 17), Con (n = 18), RG30_L (n = 15), RG30_H (n = 18), DZP (n = 23). The scale bar represents 200 µm.

The DRN is a key brain region involved in sleep regulation, and serotonin, a neurotransmitter, plays a crucial role in this process. The 5-HT concentration in the brain was measured in the RG30-treated groups (Fig 6). The Con group had significantly decreased 5-HT levels in the DRN relative to the Nor group (*p* < 0.05; Fig 6). The DZP group had significantly increased 5-HT levels in the DRN relative to the Con group (*p* < 0.05; Fig 6). However, the RG30-treated groups exhibited no significant differences in 5-HT levels compared with the Con group.

## Discussion

In this study, RG was selected and assessed for sedative properties. The findings revealed that RG30 exhibits higher binding activity to GABA_A_ receptors than to 5-HT_2C_ receptors, implying a natural ligand-binding affinity for GABA_A_ receptors. RG30 exhibited a more pronounced inhibitory effect on interactions with the GABA_A_ receptor than with the 5-HT_2C_ receptor.

RG30_L administration significantly reduced total wake time, accompanied by increased NREM and total sleep time. Our findings suggest that both RG30_L and RG30_H improved sleep parameters compared to DZP. While RG30_L showed greater improvements in certain measures, these results were not significantly different from RG30_H and require further investigation to determine optimal dosing. Moreover, RG30_H administration resulted in a substantial decrease in orexin levels in the LH, suggesting the potential of RG30 to enhance sleep patterns by reducing arousal. GABA_A_ receptor level was also significantly increased in the VLPO, suggesting that RG30 may enhance sleep patterns by modulating the GABAergic system in sleep-related brain regions, particularly within the VLPO. However, there was no significant difference in 5-HT levels in the DRN between the RG30-treated groups.

In this study, RG30 revealed a dose-dependent binding affinity, particularly towards the GABA_A_ receptor. These findings underscore the intrinsic natural ligand-binding affinity of RG30 to the GABA_A_ receptor, providing valuable insights for the swift screening and development of novel drug candidates.

EEG is used to measure the brain’s electrical activity by attaching electrodes to the scalp to record brain waves. Patterns and changes in brain activity were analyzed to assess different sleep stages [13]. In NREM sleep, delta brainwave predominance is a characteristic of deep sleep. In REM, a state marked by rapid eye movements and heightened neural activity, theta and beta brainwaves are prominent. Wakefulness is characterized by high-frequency, low-amplitude EEG recordings [14]. Pentobarbital, a central nervous system depressant, has calming and sleep-inducing effects [15].

Consequently, the characteristic EEG patterns representing different sleep stages undergo alterations. During NREM, the administration increases the brainwave cycle and decreases it during wakefulness. These EEG changes provide crucial information for evaluating the effects of drugs on sleep and wakefulness [16]. Similarly, in the present study, pentobarbital administration to rats decreased wakefulness and increased NREM sleep time. These results suggest that pentobarbital plays a role in stabilizing sleep patterns by restoring wakefulness and NREM sleep duration. This observation aligns with the notion that pentobarbital contributes to the modulation of sleep stages, similar to its role in altering EEG patterns [17]. In the current study, RG treatment decreased wake time and increased total sleep time and NREM sleep. Therefore, RG can improve pentobarbital-induced sleep.

The exploration of sleep mechanisms was the cornerstone of our study. Based on the receptor binding results, we focused on unraveling sleep-related signaling pathways, including GABA_A_ receptors, orexin, and 5-HT mechanisms. Additionally, we aimed to elucidate the effects of RG30 on sleep improvement and maintenance.

The LH has emerged as a pivotal brain region, intricately regulating the sleep–wake cycle. Orexin, synthesized within the LH, is crucial for modulating the sleep-arousal continuum [18]. Its dysregulation, either through insufficient or excessive activation of orexin, has been implicated in the manifestation of sleep disorders and related issues. LH orchestrates sleep-explosive activity, and disruptions in orexin function may disturb the delicate balance between sleep and wakefulness, contributing to a spectrum of sleep-related problems. This underscores the significance of investigating the roles of LH and orexin in understanding and addressing sleep-related disturbances. We found that RG30 decreased the number of orexin-positive cells in the LH region. The RG30 may decrease orexin levels in the LH, reducing arousal and potentially enhancing sleep quality.

The VLPO in the basal forebrain plays a central role in sleep regulation. Its activation involves the modulation of GABA_A_ receptors, which influence the initiation and maintenance of sleep [19]. This dynamic interaction significantly contributes to establishing and maintaining normal sleep patterns, orchestrating transitions between deep sleep stages and REM sleep. The activation of GABA_A_ receptors is a key determinant of sleep quality and plays a crucial role in sleep-inducing medications and tranquilizers, thereby enabling fine-tuning of sleep patterns.

Pentobarbital, a barbiturate, binds to GABA_A_ receptors [20]. The activation of GABA_A_ receptors coupled with pentobarbital leads to sleep-inducing effects on the central nervous system [21]. We administered RG30 in a pentobarbital-induced sleep animal model to investigate whether RG30 is associated with the GABAergic system and enhances sleep activity. We observed changes in GABA_A_ receptors in the VLPO. Consequently, by observing an increased number of GABA_A_ receptors in the VLPO, we confirmed that RG30 enhanced pentobarbital-induced sleep activity.

Based on the sleep-improving effects of RG30 in rats, we aimed to introduce RG30 as a potential human sleep aid or treatment for sleep disorders. In this study, we utilized receptor-binding assays and analyzed representative parameters, such as EEG, orexin, GABA_A_ receptors, and 5-HT, to investigate the effects of RG30 on sleep in a rat model. However, these analyses only confirmed the partial effects of RG30. In a pentobarbital-induced sleep model, RG30 was found to reduce wakefulness and increase total sleep duration. Notably, the efficacy of RG30 was enhanced compared with that of the conventional sleep-aid, DZP.

According to a previous study in mice, RG was suggested to counteract caffeine-induced sleep disturbances, such as sleep onset delay and decreased sleep duration [9]. In our study, we found that RG30 maintained a longer and more stable sleep duration in rats induced by pentobarbital. Despite these documented benefits, there has been no research on the sleep-promoting mechanism mediated by RG30 [9]. Future investigations should aim to elucidate the underlying mechanisms by examining the involvement of specific receptor subtypes, such as GABA_A_ and 5-HT_2C_ receptors. Moreover, comprehensive analyses of neurotransmitter alterations—including GABA, serotonin, and melatonin in sleep-regulating brain regions, as well as characterization of the associated neural circuits, are warranted. Molecular and in vitro studies may further aid in identifying the key targets and signaling pathways responsible for the observed effects. Furthermore, the orexin and GABA_A_ receptor expression levels decreased and increased, respectively; however, there were no significant changes in the 5-HT expression levels. The specific mechanisms underlying the effects of RG30 on wake/REM/NREM sleep have not yet been determined, necessitating further research.

## Conclusion

The sedative effect of RG30 may be associated with the activation of central GABAergic systems, as indicated by the increased affinity of GABA_A_ receptor binding and elevated levels of GABA_A_ receptors observed in the rat brain. Thus, RG30 may be a promising novel compound in natural products for inducing sleep.
